# The Fanconi Anemia DNA Repair Pathway Is Regulated by an Interaction between Ubiquitin and the E2-like Fold Domain of FANCL*

**DOI:** 10.1074/jbc.M115.675835

**Published:** 2015-07-06

**Authors:** Jennifer A. Miles, Mark G. Frost, Eilis Carroll, Michelle L. Rowe, Mark J. Howard, Ateesh Sidhu, Viduth K. Chaugule, Arno F. Alpi, Helen Walden

**Affiliations:** From the ‡ Protein Structure and Function Laboratory, Lincoln's Inn Fields Laboratories of the London Research Institute, Cancer Research, United Kingdom, 44 Lincoln's Inn Fields, London WC2A 3LY, United Kingdom,; § Medical Research Council Protein Phosphorylation and Ubiquitylation Unit, College of Life Sciences and; ¶ Scottish Institute for Cell Signalling, College of Life Sciences, University of Dundee, Dow Street, Dundee DD1 5EH, United Kingdom, and; ‖ Protein Science Group, School of Biosciences, University of Kent, Canterbury, Kent CT2 7NZ, United Kingdom

**Keywords:** DNA repair, E3 ubiquitin ligase, isothermal titration calorimetry (ITC), nuclear magnetic resonance (NMR), ubiquitin, structure

## Abstract

The Fanconi Anemia (FA) DNA repair pathway is essential for the recognition and repair of DNA interstrand crosslinks (ICL). Inefficient repair of these ICL can lead to leukemia and bone marrow failure. A critical step in the pathway is the monoubiquitination of FANCD2 by the RING E3 ligase FANCL. FANCL comprises 3 domains, a RING domain that interacts with E2 conjugating enzymes, a central domain required for substrate interaction, and an N-terminal E2-like fold (ELF) domain. The ELF domain is found in all FANCL homologues, yet the function of the domain remains unknown. We report here that the ELF domain of FANCL is required to mediate a non-covalent interaction between FANCL and ubiquitin. The interaction involves the canonical Ile44 patch on ubiquitin, and a functionally conserved patch on FANCL. We show that the interaction is not necessary for the recognition of the core complex, it does not enhance the interaction between FANCL and Ube2T, and is not required for FANCD2 monoubiquitination *in vitro*. However, we demonstrate that the ELF domain is required to promote efficient DNA damage-induced FANCD2 monoubiquitination in vertebrate cells, suggesting an important function of ubiquitin binding by FANCL *in vivo*.

## Teaser

**Background:** The E3 ligase FANCL monoubiquitinates FANCD2 in a critical step in the repair of DNA interstrand crosslinks.

**Results:** FANCL binds ubiquitin non-covalently via its N-terminal E2-like fold.

**Conclusion:** Monoubiquitination of FANCD2 is regulated via a non-covalent interaction between FANCL and ubiquitin.

**Significance:** This interaction represents an additional layer of regulation of the Fanconi Anemia pathway, and a targetable interface.

## Introduction

Fanconi Anemia (FA)^3^ is an autosomal recessive or X-linked inherited childhood disorder, characterized by bone marrow failure and a high incidence of cancer (1, 2). Biallelic mutations in any of 16 currently identified FA genes results in failure to repair DNA interstrand crosslinks (ICL), which can occur during replicative stress and upon encountering endogenous genotoxins (3–9).

A key step in DNA ICL repair is the site-specific monoubiquitination of FANCD2 at Lys561 (10), which leads to the recruitment of downstream repair factors. This monoubiquitination event is carried out by FANCL, the RING E3 ligase subunit of the FA core complex (11, 12). In vertebrates, the FA core complex comprises 9 proteins: FANCA, FANCB, FANCC, FANCE, FANCF, FANCG, FAAP100, FAAP20, and FANCL, reviewed in Ref. 13. Recent studies have shown that the core complex members FANCB, and FAAP100 are required for full ubiquitin ligase activity in cells (14, 15). Previous work has also suggested that FANCL is required for core complex assembly (16). Intriguingly, while FANCL as the functional ligase subunit is conserved in invertebrates, there are no identifiable homologs of FANCB or FAAP100 in lower eukaryotes. Indeed the only additional core complex component found in an invertebrate is FANCE in *Dictyostelium* (17, 18).

The crystal structure of full-length FANCL revealed 3 domains (19), an N-terminal E2-like fold (ELF) domain, a central double RWD (DRWD) domain, and a C-terminal RING domain. The RING domain serves as an E2-conjugating enzyme recruitment module, being necessary and sufficient for the interaction with Ube2T, the E2 for the FA pathway (20–22). The DRWD domain harbors the substrate binding site (19, 20, 23). However, the ELF domain has no known function and makes no contacts with the other domains of FANCL (19). The ELF domain is found in all FANCL homologues, and is conserved across species. Therefore, we sought to establish the function of the ELF domain. We report here a previously undetected non-covalent interaction between the ELF domain of FANCL and ubiquitin. We use detailed biochemical and structural studies to characterize the interaction, and find that it is mediated via a functionally conserved patch on ELF and the hydrophobic patch of ubiquitin. Furthermore we show that the interaction is neither catalytic, nor required for complex formation, but is required for efficient FANCD2 monoubiquitination in cells.

## Experimental Procedures

### 

#### 

##### Protein Expression and Purification

All mutants were generated with the QuikChange site-directed mutagenesis kit (Stratagene). *Drosophila melanogaster* FANCL constructs and DRWD-RING (105–431) were expressed and purified as described previously (19).

The ELF domain (1–105) was cloned into the pET SUMO vector. Luria Broth (LB) growth medium cultures were grown at 37 °C until *A*_600_ 0.6–0.8. After reducing the temperature to 16 °C, cells were grown to *A*_600_ 0.8 before inducing with 250 μm isopropyl β-d-1-thiogalactopyranoside (IPTG). Cells were harvested in 500 mm NaCl, 100 mm Tris, pH 8.0, 250 μm Tris(2-carboxyethyl)phosphine (TCEP), sonicated and clarified before affinity purification using Ni-NTA resin (Qiagen) and overnight cleavage with the SUMO protease, Ulp1, in 50 mm NaCl, 100 mm Tris, pH 8.0, 250 μm TCEP. The flow through was loaded onto a Q Sepharose HP in 50 mm NaCl, 100 mm Tris, pH 8.0, 250 μm TCEP and eluted with a gradient including 500 mm NaCl. The purified ELF domain was flash frozen and stored in 50 mm Tris, pH 8.0, 200 mm NaCl, 250 μm TCEP, and 5% (*v*/*v*) glycerol at −80 °C.

Human ubiquitin was cloned into pRSF Duet-1 vector with no purification tag. Luria Broth (LB) growth medium cultures were grown at 37 °C until *A*_600_ 0.5, at which point expression was induced with 500 μm IPTG for 4 h. Cells were harvested and resuspended in PBS buffer before repelleting and flash freezing in liquid nitrogen. The pellets were resuspended in 1 mm EDTA, 25 mm Tris, pH 8.0 before sonication and clarification. The clarified lysate was filtered and loaded onto Q Sepharose HP (GE Healthcare) anion exchange column equilibrated 25 mm Tris, pH 8.0, 1 mm EDTA. The ubiquitin came off in the unbound fraction, before further purification with size exclusion chromatography 250 mm NaCl, 100 mm Tris, pH 8.0 and subsequent storage at −80 °C.

For His-ubiquitin, human ubiquitin was cloned into the pRSF Duet-1 vector with a N-terminal Hexa-His tag. Expression was induced with 0.5 mm IPTG at *A*_600_ 0.5 followed by batch affinity purification on Ni-NTA (Qiagen) as above. His-ubiquitin was eluted with 300 mm imidazole and further purified by size exclusion chromatography. All interaction experiments are done using monoubiquitin.

*Xenopus laevis* STREP-FANCD2 cDNA was a kind gift from P. Knipscheer and J. Walter. We expressed and purified the protein as previously described (24). Briefly, Hi5 cells were infected with baculovirus, and the protein was purified on FLAG resin before elution with 3× FLAG peptide and stored with 5% glycerol at −80 °C.

##### Xenopus tropicalis

FANCL was expressed and purified as previously described (21).

##### Pull-down Assays

In a total reaction volume of 1 ml containing assay buffer, 500 mm NaCl, 100 mm Tris, pH 8.0, 250 μm TCEP and 10 μm ZnCl_2_, *Drosophila* FANCL, FANCL L81R, ELF, DRWD-RING, *Xenopus* FANCL, or FANCL N72R (all 250 nm) was added with an excess of His-ubiquitin (1 μm), His-Ube2T (500 nm) or His-Ube2T-Ub (500 nm). Reactions were left to bind for 1 h on ice. 100 μl of Ni-NTA-agarose (Qiagen) was equilibrated in assay buffer and added to the 1 ml reaction and left on a roller at 4 °C for 1 h. Samples were washed with 10 ml of assay buffer and the agarose resuspended in 100 μl of assay buffer. 50 μl of 2× SDS buffer was added before the samples were boiled. 10 μl of the samples was loaded onto SDS page gel and subjected to Western blotting and probed with appropriate antibodies. Anti-ubiquitin antibody was purchased from DAKO. Anti-*Drosophila* FANCL and anti-human DRWD antibodies were raised from recombinant proteins by Pettingill Technology Ltd. The anti-His antibody was purchased from GE Healthcare.

##### NMR Spectroscopy and ELF Assignment

The *Drosophila* ELF domain and ubiquitin were isotopically labeled by overexpression in minimal medium enriched with [^15^N]ammonium sulfate and [^13^C]glucose. For NMR samples the ELF domain and ubiquitin were buffer exchanged into 50 mm NaCl, 20 mm sodium phosphate, pH 6.5. Susceptibility matched NMR tubes (Shigemi, US) were used at all stages. Sample volumes of 330 μl were placed in Shigemi BMS-005V tubes and included 10% D_2_O *v*/*v*. The majority of NMR experiments were carried out at 600 MHz using a VarianUnity INOVA equipped with a 5 mm HCN z-pulse field gradient probe. Chemical shift referencing was based on the position of the water resonance with the exact value being related to the known relationship of the H_2_O resonance with temperature. Unless otherwise stated, all NMR experiments were solvent suppressed to reduce the water signal using WATERGATE that was typically obtained using gradient field strengths of 40–50 G cm^−1^. All NMR datasets were acquired at 25 °C.

All NMR data processing utilized NMRPipe (25) and analyzed using the CCPN analysis package (26). Most resonances were successfully assigned manually without ambiguity. Backbone chemical shift assignments were achieved for the ELF domain using ^1^H-^15^N HSQC, CBCA(CO)NH, CBCANH, ^15^N-NOESY-HSQC, and ^15^N-TOCSY-HSQC experiments. Previous amide chemical shift assignments for human ubiquitin were obtained from the VLI Research, Inc. Web site and were used to assign resonances in the ubiquitin ^1^H-^15^N HSQC.

##### NMR Titrations

All titration experiments were recorded in 50 mm NaCl, 20 mm sodium phosphate, pH 6.5. The ^15^N-labeled domain (wild type ELF or ubiquitin) was at 0.6 mm in the sample, while the binding partner was titrated in at different molar ratios (10:1, 7:5:1, 5:1, 2.5:1, 1:1). All ^15^N-^1^H HSQC were recorded for 30 min.

##### Isothermal Titration Calorimetry (ITC)

Kinetic information of the *Drosophila* ELF domain binding interactions with ubiquitin was established using the ITC200 microcalorimeter (MicroCal, Northampton, MA). Sample buffers were 50 mm Tris, pH 8.0, 100 mm NaCl. Experiments were carried out at 30 °C. The syringe contained 40 μl of 2.5 mm ubiquitin, with 2.5 μl injections every 200 s. The cell contained 205 μl of 230 μm ELF domain. Cell concentrations were adjusted to a 1:1 stoichiometric interaction and Microcal Origin software version 7.0 was used to determine the dissociation constants (*K_d_*). All measurements were repeated at least twice.

##### DT40 Cell Studies

*FANCL*-deficient DT40 cells (fancl^−/−^) and fancl^−/−^ complemented with TAP-tagged wild-type FANCL (TAP-FANCL) were described recently (27). Point mutations L7A, D78A, D78R, L79A, and V80A were generated by site-directed muagenesis (QuickChange II XL site-directed mutagenesis kit; Stratagene). DT40 cell transfections, DT40 subfractionation, Superpose 6 gel size chromatography, and FANCD2 immunoblot analyses were described previously (27). To assess TAP-FANCL interaction with ubiquitin, fancl^−/−^ cells expressing either TAP-FANCL or TAP-FANCL (L7A, D78R, L79A) were lysed in TAP-buffer (50 mm Tris, pH 8.0, 200 mm NaCl, 0.5 mm EDTA, 0.1% Nonidet P-40, and protease inhibitor mixture (Roche Molecular Biochemicals)). TAP-tagged FANCL variants were immunoprecipitated from 2 mg lysates with 50 μl of IgG-coupled Sepharose (GE Healthcare) and washed extensively with Ub-binding buffer (UbBB) (50 mm Tris, pH 7.4, 150 mm NaCl, 0.1% Nonidet P-40, 2 mg/ml BAS). TAP-FANCL Sepharose beads were homogenized in UbBB and incubated with 10 mg/ml HA-tagged wild type or I44A-mutated ubiquitin for 2 h at 4 °C. Beads were washed with wash buffer (50 mm Tris, pH 7.4, 250 mm NaCl, 0.2% Nonidet P-40). Bound ubiquitin was analyzed by immunoblotting with anti-HA (Bethyl Laboratories).

##### Ube2T Autoubiquitination Assay

100 μl reactions were setup containing 3 μm His-Ube2T, 227 nm E1, 28 μm His-ubiquitin with either 9 μm of E3 (WT *Drosophila* FANCL, L81R *Drosophila* FANCL, or *Drosophila* DRWD-RING) or water in a buffer containing 5 mm ATP, 50 mm Tris, pH 7.5, 100 mm KCl, 0.5 mm DTT, and 2 mm MgCl_2_. Subsequently, 10 μl samples were removed from each assay at 0, 15, 20, and 30 min, and arrested with 10 μl LDS buffer (Invitrogen) containing 400 mm β-mercaptoethanol (β-ME). Half of each sample was subjected to Western blot analysis using an anti-His antibody (GE Healthcare).

##### Charging Ube2T

Charged Ube2T was prepared by adapting the method described in Ref. 28. Human His-Ube2T C24S and tag-free ubiquitin G76C were purified to homogeneity as before, albeit retaining the His-tag on Ube2T. These were then dialyzed against 50 mm sodium borate, pH 8.0, 1 mm TCEP overnight. The proteins were mixed for 15 min on ice with His-Ube2T at 330 μm and ubiquitin at 1 mm. A stock solution of 1,3 dichloroacetone (Sigma) was prepared in dimethylformamide at 20 mm. This was added to the mixed proteins at 0.8 mm and the samples were left rolling at 4 °C for 1 h. The reaction was quenched with 10 mm β-ME for 1 h. The sample was then loaded onto an SD75 16/60 column (GE Healthcare) and purified, and the protein stored at −80 °C with 10% glycerol.

##### Monoubiquitination of Xenopus FANCD2

Ubiquitination reactions were performed at 30 °C in a 50 mm Tris, pH 7.5, 100 mm KCl, 2 mm MgCl_2_, 5% (*v*/*v*) glycerol, and 0.5 mm dithiothreitol (DTT) buffer system. Reactions contained 25 nm of recombinant human E1, 0.5 μm of Ube2T, 1 μm of indicates E3 species, 0.5 μm of *Xenopus FANCD2*, and 2 mm ATP in 20 μl of final reaction volume.

Complete ubiquitination profile was analyzed using fluorescently labeled ubiquitin (Ub^800^). Ubiquitin (residues 2–76) was expressed and purified bearing a GPL*C*GS overhang at the N terminus. The cysteine residue in the overhang was targeted for site-specific incorporation of a DyLight™ 800 Maleimide (Life Technologies) dye following the manufacturer's protocol. Labeled species was further subject to cation exchange chromatography and stored at −20 °C as single-use aliquots. All ubiquitination reactions contained 2 μm of Ub^800^ and were terminated by boiling with LDS loading buffer. The samples were resolved by SDS-PAGE and analyzed by direct fluorescence monitoring using Li-COR® Odyssey Infrared Imaging System. Integrated intensities of *FANCD2* ubiquitination from five independent experiments were obtained using Image Studio™ (Odyssey) imaging software and plotted using GraphPad Prism®.

## Results

### 

#### 

##### FANCL Binds Ubiquitin Non-covalently

**FIGURE 1.**
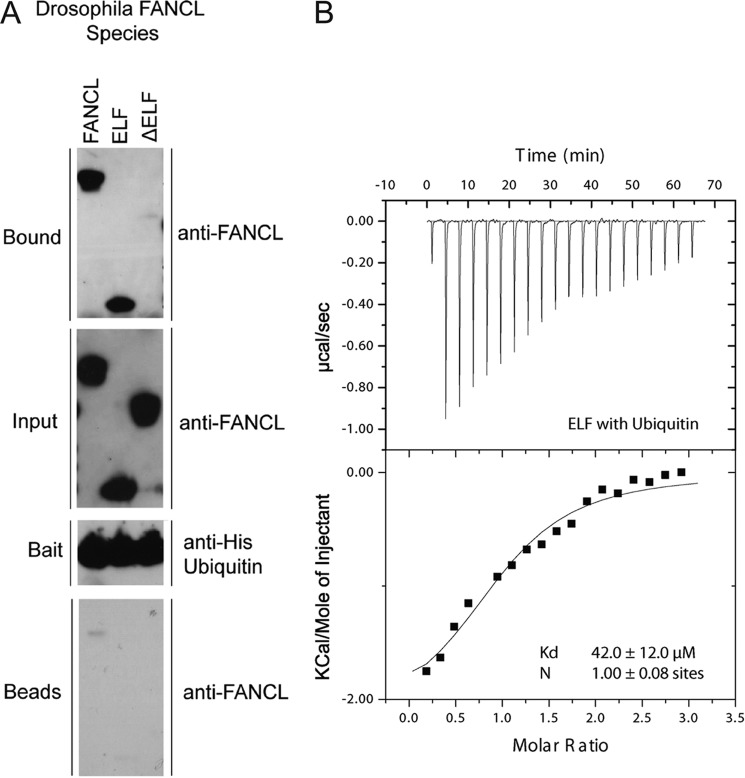
**FANCL binds ubiquitin via the N-terminal E2-like fold.** *A*, pull-down of FANCL species by ubiquitin shows that ubiquitin binding is mediated by the ELF domain. Each experiment is probed with anti-His-ubiquitin and anti-FANCL antibodies, with the input, bait, and beads controls indicated. *B*, isothermal titration calorimetry curve showing binding of the ELF domain to ubiquitin. Dissociation constant and stoichiometry of the interaction are indicated.

The ELF domain of FANCL shares significant structural homology with E2-conjugating enzymes (19). E2s form a catalytic intermediate with ubiquitin via a thioester between the catalytic cysteine and the C terminus of ubiquitin (29). The ELF domain does not possess a catalytic cysteine. However, an additional feature of E2s is that they can also interact non-covalently with ubiquitin (30, 31). Therefore we hypothesized that the ELF domain of FANCL might interact with ubiquitin in a similar manner. To test this hypothesis, we performed a pull-down binding assay using 6× His-tagged ubiquitin as bait (Fig. 1*A*). His-ubiquitin pulls down both full-length *Drosophila* FANCL and the isolated ELF domain. In contrast, FANCL lacking the ELF domain (ΔELF) is not pulled down. To further characterize the interaction between the ELF domain and ubiquitin, we measured the affinity of binding using isothermal titration calorimetry (ITC), and determined a dissociation constant of 42 ± 12.0 μm (Fig. 1*B*).

##### A Surface-exposed Patch on the ELF Domain Interacts with a Hydrophobic Patch on Ubiquitin

**FIGURE 2.**
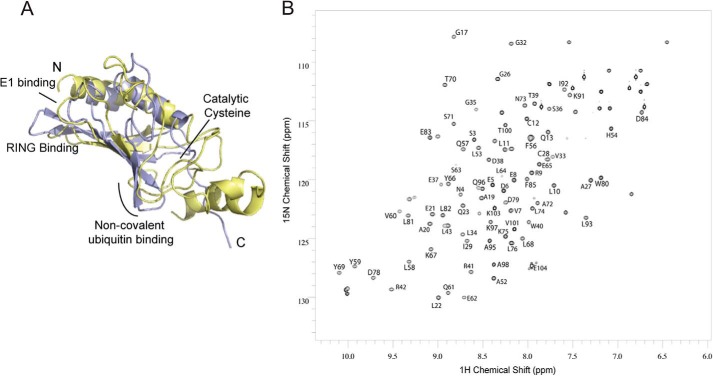
**Structural assignment of the *Drosophila* ELF domain.** *A*, superposition of the ELF domain from *Drosophila* FANCL in *blue* (PDB 3K1L) (19) with the E2 Ube2L3 in *yellow* (PDB 1FBV) (48). E2 protein-protein interaction surfaces indicated, as is the position of the catalytic cysteine of Ube2L3. *B*, assignment of the *Drosophila* ELF domain ^1^H-^15^N HSQC. The cross-peaks in the ^1^H-^15^N HSQC were assigned to residues in the primary sequence of the *Drosophila* ELF domain.

**FIGURE 3.**
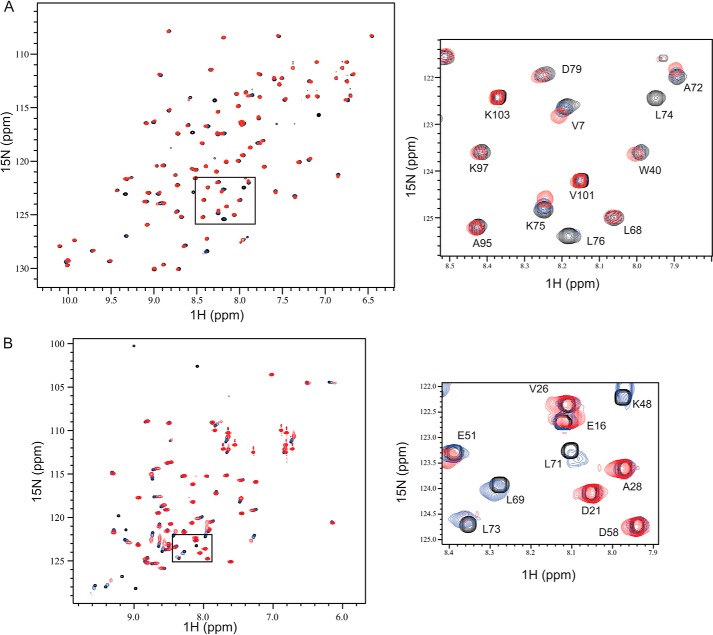
**Reciprocal titrations of FANCL ELF domain and ubiquitin indicate interaction between both proteins.** *A*, ^15^N-^1^H HSQC of the ^15^N labeled ELF domain during titration of wild type ubiquitin. Wild type ELF domain spectra are denoted in *black*, with 5:1 ELF to ubiquitin in *blue* and 1:1 in *red*. The *box* is a zoom of a portion of the spectra. *B*, ^15^N-^1^H HSQC of ^15^N-labeled ubiquitin during titration of wild-type ELF. Wild type ubiquitin spectra are in *black*, with 5:1 ubiquitin to ELF in *blue* and 1:1 in *red*. The *box* is a zoom of a portion of the spectra.

**FIGURE 4.**
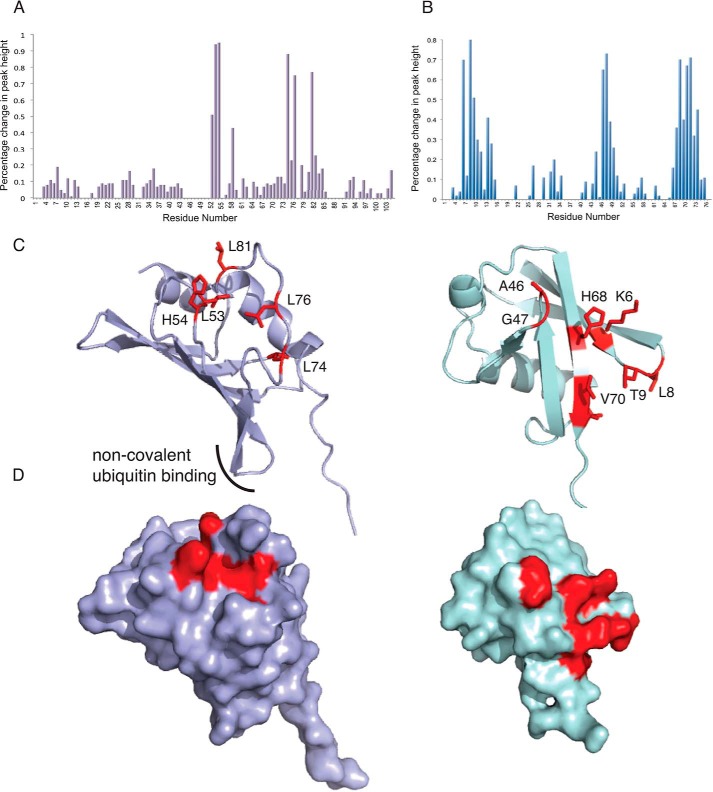
**A solvent-exposed patch on the ELF domain interacts with the hydrophobic Ile44 patch of ubiquitin.** *A*, graphical representation of the shifts in cross-peaks in the spectra of the ELF domain upon titration of unlabeled ubiquitin. *B*, graphical representation of the shifts in cross-peaks in the spectra of ubiquitin upon titration of unlabeled ELF domain. The *y* axis represents the percentage decrease in cross-peak height for each residue between the wild-type ^1^H-^15^N HSQC and the ^1^H-^15^N HSQC recorded with 5:1 ^15^N-labeled protein. *C*, ribbon diagram of the *Drosophila* ELF domain (in *purple*) and ubiquitin (in *blue*) with residues involved in binding highlighted in *red. D*, surface representations of the *Drosophila* ELF domain (in *purple*) and ubiquitin (in *blue*) with residues involved in binding shown in *red*.

**FIGURE 5.**
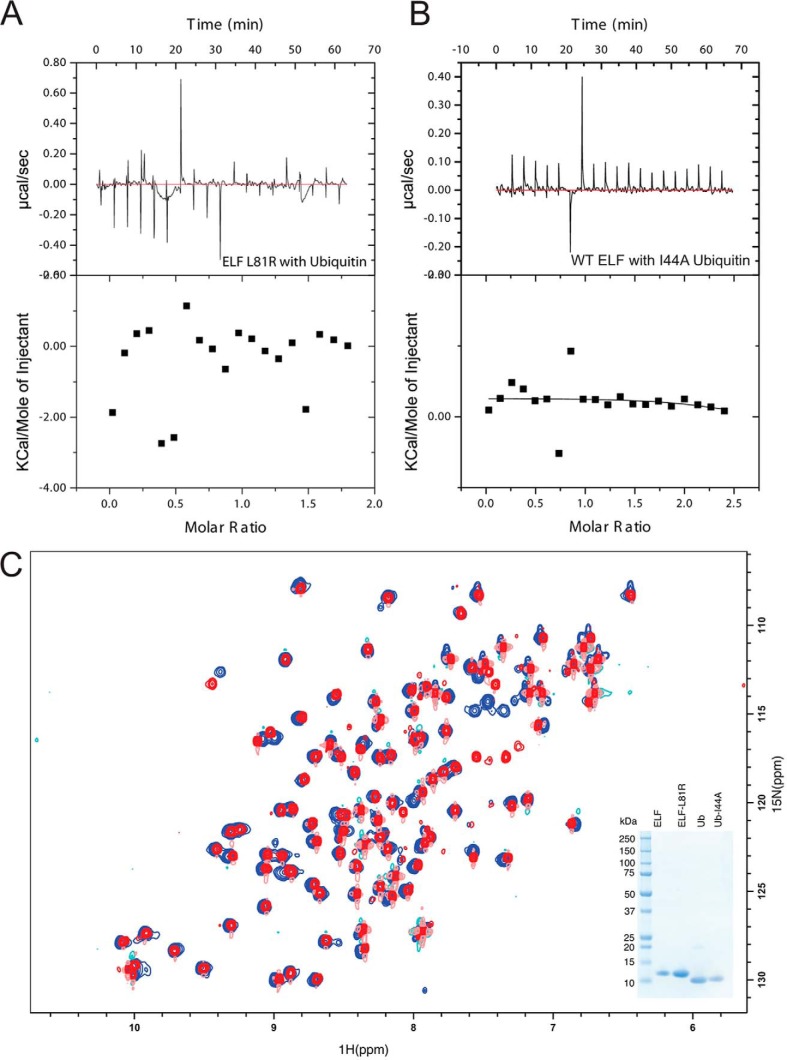
**Mutation of the ELF domain abolishes binding.** *A*, ITC curves showing lack of interaction between ubiquitin and L81R ELF domain. *B*, ITC curves showing lack of interaction between the ELF domain and I44A ubiquitin. *C*, ^1^H-^15^N HSQC spectra of wild-type DmELF domain (*blue*) overlaid with ^1^H-^15^N HSQC spectra of DmELF-L81R (*red*). The overlay shows the structure, fold, and stability of both proteins are comparable. The *inset* shows a Coomassie-stained gel of the proteins used in these experiments.

We next wanted to understand the molecular determinants of the interaction between FANCL and ubiquitin. E2s bind ubiquitin non-covalently via a backside interaction that involves residues from the loop connecting strands β2 and β3 (30) (Fig. 2*A*). The dissociation constant between ubiquitin and the ELF domain suggests that crystallization of the complex would prove challenging. Indeed, despite extensive efforts, we were unable to obtain high-resolution diffracting crystals. Therefore, to understand the mode of ubiquitin binding by the ELF domain and whether it is similar to that seen in E2s, we set out to map the interacting surfaces using Nuclear Magnetic Resonance (NMR) spectroscopy. For both structural studies and ITC, milligram quantities of high-quality protein are required. The mammalian and vertebrate homologues of FANCL are not amenable to large scale soluble expression (20, 23); therefore, we used the more soluble invertebrate ELF domain from *Drosophila*, which shares ∼65% sequence similarity (19% identity) with the human ELF domain (19). First, we determined the solution structure of the ELF domain. Two-dimensional ^15^N-^1^H HSQC NMR of the ^15^N-labeled ELF domain yielded clear and resolved spectra, with excellent chemical shift dispersion, characteristic of a folded globular domain. We unambiguously assigned 76 out of 104 ELF residues using triple-resonance backbone datasets (Fig. 2*B*). Once we had determined the positions of each residue of the ELF domain in the spectra, we then titrated in increasing amounts of ubiquitin and recorded changes in the two-dimensional ^15^N-^1^H HSQC. Upon addition of ubiquitin, resonances were broadened to the extent that they were no longer visible, indicating a specific but transient interaction between the proteins (Fig. 3*A*). We then performed the reciprocal experiments by titrating increasing wild type ELF domain into ^15^N-labeled ubiquitin, and identified the binding site on ubiquitin (Fig. 3*B*). The interaction surface on the ELF domain involves a surface comprising residues Leu-53, His-54, Leu-74, Leu-76, and Leu-81 (Fig. 4, *A* and *B*). The interaction surface on ubiquitin is the Leu8-Ile44-Val70 central hydrophobic patch commonly recognized by ubiquitin-binding proteins (Fig. 4, *A* and *B*) (32). These results reveal a novel interaction surface on the ELF domain. This surface is not a relic of the E2-like fold, as it does not coincide with the predicted surface upon overlaying the structures (Figs. 2*A* and 4, *C* and *D*). To assess the requirement for residues in the interaction surfaces, we sought to validate our structural insights. We mutated residues involved in the binding, and assayed the resulting proteins for interaction using ITC. The ELF domain point mutant L81R completely abolishes binding (Fig. 5*A*), as does the ubiquitin mutant I44A (Fig. 5*B*). To test whether the leucine to arginine mutation on the exposed solvent-accessible surface of the ELF domain disrupts the folding of the domain, we performed two-dimensional ^15^N-^1^H HSQC NMR of the ^15^N-labeled L81R-ELF domain. These experiments yielded clear and resolved spectra, with excellent chemical shift dispersion, characteristic of a folded globular domain, with small changes compared with the wild-type spectra, consistent with a well-folded, stable, mutant protein (Fig. 5*C*).

##### Ub Binding Is a Conserved Function of Vertebrate and Invertebrate ELF Domain

**FIGURE 6.**
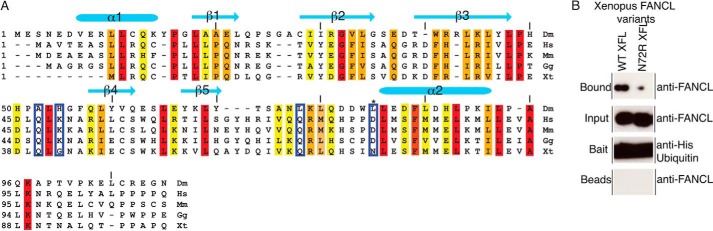
**Ubiquitin binding is conserved in vertebrates.** *A*, structure-based alignment of the ELF domain from various species of FANCL: *Drosophila melanogaster* (Dm), human (Hs), mouse (Mm), chicken (Gg), *Xenopus tropicalis* (Xt), and *Danio rerio* (Dr). Conserved residues are shaded *red*, conservative substitutions in *orange*, semi-conservative substitutions in *yellow*. Residues involved in ubiquitin-binding are *boxed*, and the Leu-81/Asn-72 residue is marked with an *asterisk*. Structural elements are included above the sequence. *B*, pull-down of *Xenopus Tropicalis* FANCL by ubiquitin shows that ubiquitin binding is conserved. Each experiment is probed with anti-His-ubiquitin and anti-FANCL antibodies, with the input, bait and beads controls indicated.

The residues in the ELF domain that are important for ubiquitin binding are not well conserved (Fig. 6*A*). However, ubiquitin-interacting proteins and motifs such as CUE domains, UBAs, UIMs, and MIUs often share very little sequence homology yet retain functional homology (33). We therefore wanted to determine whether the function of ubiquitin binding is conserved in vertebrate FANCL homologs. We and others had difficulties to make wild type full-length human FANCL, therefore we turned to the *Xenopus* system. Indeed. *Xenopus tropicalis* FANCL recapitulates ubiquitin binding (Fig. 6*B*). Mutation of Asn-72, corresponding to Leu-81 in the *Drosophila* protein, results in almost a complete loss of interaction with ubiquitin, indicating that the same region in FANCL is required for interaction with ubiquitin (Fig. 6*B*), and that the interaction is conserved between species.

##### FANCL-Ubiquitin Binding Does Not Enhance the Interaction with Charged Ube2T or Aid Ube2T Discharge

**FIGURE 7.**
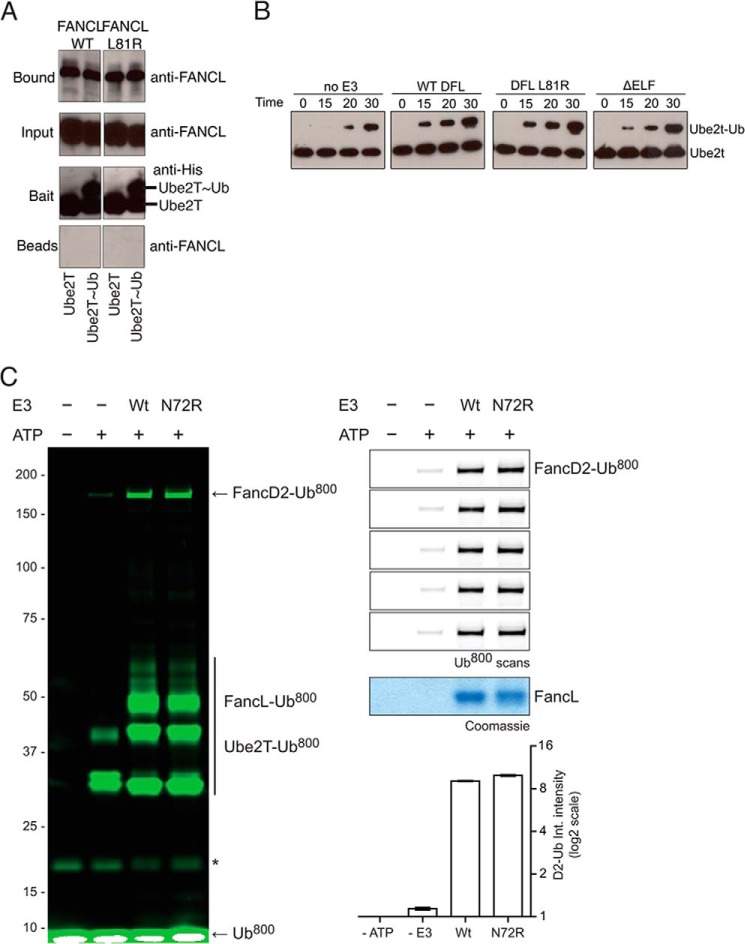
**Ubiquitin binding is not required for E2 recognition.** *A*, pull-down analysis of the interaction between wild type and L81R *Drosophila* FANCL and human Ube2T or Ube2T-Ub. Both FANCL species bound Ube2T and Ube2T-Ub to the same extent. *B*, Western blot analysis of Ube2T autoubiquitination in the absence and presence of *Drosophila* FANCL WT, L81R, and ΔELF species. All variations of E3 were able to successfully stimulate discharge of ubiquitin from Ube2T onto itself. *C*, in-gel fluorescence analysis of *in vitro* FANCD2 monoubiquitination (*left*). Ubiquitin is fluorescently labeled, with no ATP and no E3 controls, showing the modification of FANCD2. The *right panel* shown 5 independent replicates, with a Coomassie-stained loading control, and quantification of the level of FANCD2 ubiquitination (*bottom*).

Several E2-RING E3 ligase interactions are enhanced by the presence of the ubiquitin thioester bound on the E2 (34, 35). Therefore another explanation is that FANCL interaction with ubiquitin enhances the recognition of ubiquitin-charged Ube2T (Ube2T∼Ub). To test this hypothesis, we generated a stable Ube2T∼Ub ester and assayed FANCL binding via pull-down. We observed no difference in the levels of FANCL binding in a comparison between ubiquitin-charged Ube2T and uncharged Ube2T (Fig. 7*A*). Furthermore, there was no difference in Ube2T∼Ub binding when the ubiquitin binding surface of ELF was mutated. These data suggest that ubiquitin binding does not enhance FANCL's interaction with E2.

Although ubiquitin binding does not enhance interaction with Ube2T∼Ub, it may be required for efficient discharge of the ubiquitin thioester. Since Ube2T auto-monoubiquitinates (22), and FANCL has been shown to stimulate this (22), we performed a thioester discharge assay to test whether the ELF-Ubiquitin interaction is important for this. We incubated full-length *Drosophila* FANCL, FANCL L81R, or ΔELF with human Ube2T, and monitored levels of Ube2T autoubiquitination. Indeed, addition of FANCL, FANCL L81R, or ELF-deleted FANCL (ΔELF), stimulated discharge of ubiquitin to the same extent (Fig. 7*B*). This suggests that the ELF domain is neither enhancing discharge nor monoubiquitination and is thus not catalytic.

##### Ubiquitin Binding Is Not Required for Efficient FANCD2 Ubiquitination in Vitro

We next asked whether FANCL's non-covalent interaction with ubiquitin is important for FANCD2 monoubiquitination *in vitro*. Wild type FANCL supports FANCD2 monoubiquitination *in vitro* (Fig. 7*C*). Mutation of the ubiquitin-binding patch in has no effect on the levels of FANCD2 monoubiquitination, further suggesting that the ubiquitin-binding by the ELF domain is not catalytic.

##### Ubiquitin Binding by FANCL Is Required for Efficient Monoubiquitination of FANCD2 in Vertebrate Cells

**FIGURE 8.**
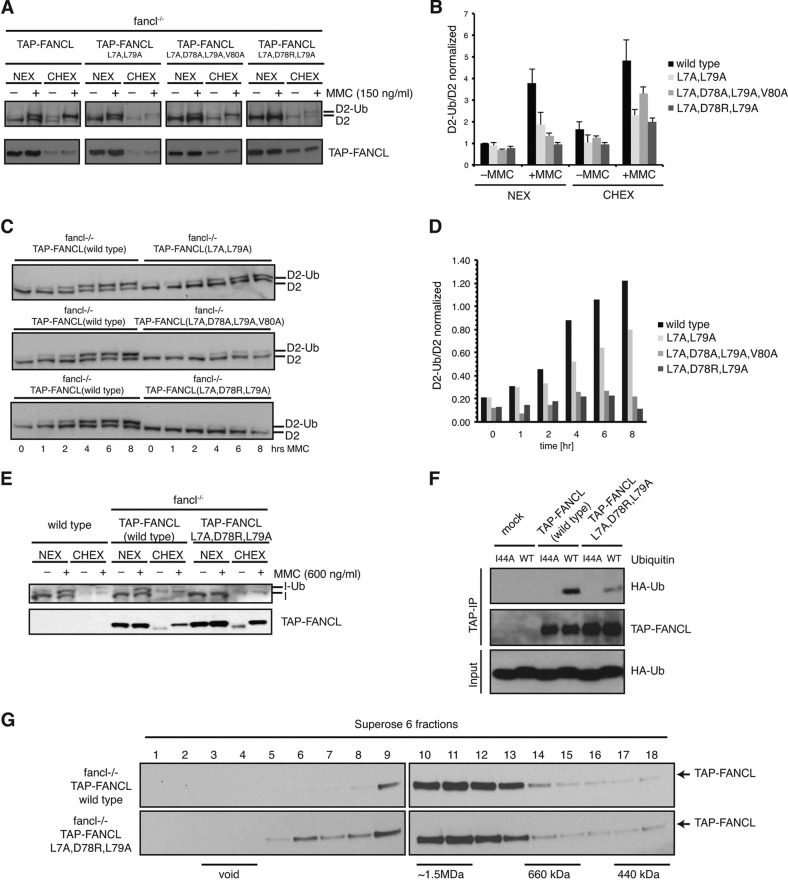
**Ubiquitin binding by FANCL is required for efficient FANCD2/FANCI monoubiquitination in vertebrate cells.** *A*, *FANCL*-deficient DT40 cells (fancl^−/−^) were complemented with TAP-tagged wild-type FANCL (TAP-FANCL) and FANCL with mutated ELF ubiquitin-binding sites (TAP-FANCL(L7A, L79A), TAP-FANCL(L7A, D78A, L79A, V80A), TAP-FANCL(L7A, D78R, L79A)). Cells were either 150 nm MMC-treated (+) or mock treated (−), and lysates were subfractionated into high salt nuclear extract (*NEX*) and soluble chromatin extract (*CHEX*). Equal total protein amount of extracts were separated on SDS-PAGE gels and analyzed by immunoblotting using anti-FANCD2 and anti-TAP antibodies. Mutations in the ELF ubiquitin-binding site perturbed MMC-induced FANCD2 monoubiquitination. D2-Ub, monoubiquitinated FANCD2; D2, unmodified FANCD2. *B*, quantitation of the various ratios of monoubiquitinated FANCD2 and unmodified FANCD2 shown in *A* using ImageJ analysis software. Standard error of the mean is given from three independent experiments. *C*, cell lines described in *A* were exposed to 150 ng/ml MMC, whole cell extract prepared after indicated times and subjected to FANCD2 immunoblot analysis. *D*, quantitation of the various ratios of monoubiquitinated FANCD2 and unmodified FANCD2 shown in *C* using ImageJ analysis software. *D*, indicated cell lines were treated with 600 nm MMC (+) or mock treated (−), fractionated as described in *A*, and analyzed by immunoplotting using anti-FANCI and anti-TAP. FANCI monoubiquitination was significantly reduced in ELF-mutated cells. *I-Ub*, monoubiquitinated FANCI; *I*, unmodified FANCI. *F*, TAP-tagged wild-type FANCL (TAP-FANCL) and ELF-mutated FANCL (TAP-FANCL [L7A, D78R, L79A]) were affinity-purified from corresponding DT40 cells with IgG-Sepharose, and incubated with either wild type HA-ubiquitin (*WT*) or I44A mutated HA-ubiquitin (I44A). Co-precipitation of the ubiquitin forms were analyzed by immunoplotting using anti-HA. Mutating the ELF domain or the ubiquitin I44 hydrophobic patch disrupted the TAP-FANCL ubiquitin interaction. *G*, TAP-FANCL and TAP-FANCL (L7A D78R L79A) high salt nuclear extracts were fractionated by Superose 6 size exclusion chromatography, and fractions were analyzed by immunoplotted using anti-TAP. Elution profiles of a 1–1.5 MDa complex were comparable between wild type FANCL and the ELF domain mutated FANCL.

Finally, we asked whether ubiquitin binding by FANCL has any relevance to FANCD2 monoubiquitination in cells. To test this hypothesis we expressed ELF domain-mutated versions of TAP-tagged FANCL in FANCL-deficient avian DT40 cells (fancl^−/−^). These FANCL variants carry combinatorial point mutations of conserved amino acids, L7A, D78A, D78R, L79A, V80A, that assemble the ubiquitin binding surface determined from *Drosophila* FANCL (Fig. 6*A*). Wild-type TAP-tagged FANCL expression promotes efficient FANCD2 monoubiquitination both in high salt nuclear extracts (NEX) and soluble chromatin extracts (CHEX) (Fig. 8). In contrast, TAP-FANCL (L7A, L79A), TAP-FANCL (L7A, D78A, L79A, V80A), and TAP-FANCL (L7A, D78R, L79A) are defective in mitomycin C (MMC)-induced monoubiquitination of FANCD2 (Fig. 8, *A* and *B*). In addition we compared time-dependent FANCD2 monoubiquitination following MMC treatment of wild-type TAP-FANCL with ELF domain mutants FANCD2 monoubiquitination following MMC treatment of wild type TAP-FANCL with ELF ubiquitin binding mutants TAP-FANCL (L7A, L79A), TAP-FANCL (L7A, D78A, L79A, V80A), and TAP-FANCL (L7A, D78R, L79A) (Fig. 8, *C* and *D*). TAP-FANCL (L7A, D78A, L79A, V80A), TAP-FANCL (L7A, D78R, L79A), and to a lesser extent TAP-FANCL (L7A, L79A), showed a significant delay in and overall reduction of FANCD2 monoubiquitination. Moreover, we observed a similar reduction of MMC-induced FANCI monoubiquitination (Fig. 8*E*).

FANCL was originally predicted to adopt a WD40-propeller fold in place of the ELF-DRWD domains (12). In a previous study based on this prediction, mutation of the predicted WD40 repeats, including a large part of the ELF domain, was found to disrupt assembly of the FA core complex (16). Since the core complex is required for efficient FANCL-catalyzed FANCD2 monoubiquitination in cells, we next assessed whether the defective FANCI/FANCD2 monoubiquitination in TAP-FANCL (L7A, D78R, L79A) expressing cells was due to disrupted ELF ubiquitin-binding rather than a destabilization of the FA core complex. We first tested TAP-FANCL ubiquitin-binding employing a cell free system. Affinity purified TAP-FANCL interacted with wild type ubiquitin, that was dependent on the ubiquitin Ile-44 patch. Mutated TAP-FANCL (L7A, D78R, L79A) however showed a significantly reduced ubiquitin binding, confirming that amino acids Leu-7, Asp-78, and Leu-79 encompass the ubiquitin interaction surface on chicken FANCL (Fig. 8*F*).

Next we assayed the formation of the FA core complexes in DT40 FANCL^−/−^ cells expressing TAP-tagged FANCL or TAP-tagged ELF patch mutant TAP-FANCL (L7A, D78R, L79A). We observed no difference in the pattern of high molecular weight complex formation (Fig. 7*A*), indicating that ubiquitin binding is not required for the stable incorporation of FANCL into the core complex. Notably, TAP-FANCL (L7A, D78R, L79A) accumulates on damaged chromatin as efficient as wild type TAP-FANCL, further supporting our data that TAP-FANCL complex formation and integrity is independent of ELF ubiquitin binding (Fig. 8*G*). Taken together, these data suggest that in vertebrate cells, FANCI/FANCD2 monoubiquitination is dependent on the FANCL interaction with ubiquitin, mediated by the ELF domain.

## Discussion

This study describes a hitherto unknown non-covalent interaction between FANCL and ubiquitin, with an affinity commonly observed in ubiquitin-protein interactions (36), that is required for efficient FANCD2 monoubiquitination. The interaction between the ELF domain of FANCL and ubiquitin is distinct from the surfaces in E2 proteins commonly used for non-covalent ubiquitin binding. This suggests that rather than being a relic of the E2 fold, ubiquitin binding is a function specific to FANCL. We define a function for the ELF domain, which is conserved among FANCL species (19), although dispensable *in vitro* for both substrate binding and catalysis of FANCD2 monoubiquitination (19–21). Our finding that the ubiquitin-binding patch of the ELF domain is required for efficient FANCD2 monoubiquitination in cells suggest that ELF domain mutations would be harmful. However, there are as yet no FA patients identified with mutations in this domain. Intriguingly, a recent study aimed at identifying breast cancer susceptibility genes reports a significant occurrence of a splice isoform of FANCL in a cohort of non-BRCA breast cancer patients (37). The isoform results in removal of residues 72–91 of the ELF domain, which encompasses the ubiquitin-interacting surface (Figs. 4*C* and 6*A*).

Our findings suggest that FANCL may be binding an unidentified ubiquitinated protein as a requisite step in the monoubiquitination of FANCD2. We propose that a potential candidate is the clamp loader, PCNA. ICL repair is a complex and multistep process, that also requires components of the translesion synthesis (TLS) pathway (9, 38, 39). In common with the FA pathway, the TLS pathway is also regulated by a site-specific monoubiquitination event. PCNA is modified at Lys164 by the ring E3 ligase, Rad18 (40–42). FANCL and Rad18 are epistatic for ICL sensitivity and repair (43), and co-depletion of FANCD2 and Rad18 does not increase cellular sensitivity to cisplatin, suggesting the proteins function in the same pathway (44). Rad18 does not monoubiquitinate FANCD2 (45). However, the E3 ligase activity of Rad18 is required for efficient loading of FANCD2 onto chromatin (44). In addition to the requirement for Rad18 activity, PCNA and FANCD2 interact in cells (46). FANCL and PCNA are also reported to interact in cells, via the central (DRWD) domain of FANCL (47). Although the monoubiquitination of PCNA appears to be critical for monoubiquitination of FANCD2, the molecular and mechanistic details of this interplay are poorly understood. We propose that the ELF domain of FANCL interacts with monoubiquitinated PCNA, and that may act as a trigger for FANCD2 monoubiquitination, as observed by Geng *et al.* (47). Potentially, monoubiquitinated PCNA reinforces the interaction between FANCD2 and FANCI. Alternatively, given that monoubiquitination of PCNA is required for accumulation of FANCA on chromatin (45), monoubiquitinated PCNA could aid in recruitment of the core complex to sites of DNA damage and/or activation of FANCL. In summary, our data provide insights into the regulation of the FA pathway, and provides the molecular details of a required interaction, potentially representing a druggable interface in the FA pathway.

## Author Contributions

J. A. M. designed and performed experiments in Figs. 1, 2, 3, 4, 5, 6, and 7. MGF performed experiments in Fig. 5, and multiple efforts to address reviewer requests. M. L. R. and M. J. H. performed experiments in Figs. 2, 3, and 4. E. C. and A. F. A. performed experiments in Fig. 8. AS assisted with NMR assignments. V. K. C. performed experiments in Fig. 7. H. W. and A. F. A. conceived the study, analyzed results and wrote the paper.

## Footnotes

3 The abbreviations used are:

FA: Fanconi Anemia
ICL: interstrand crosslinks
ELF: E2-like fold
TCEP: Tris(2-carboxyethyl)phosphine
ITC: isothermal titration calorimetry.
